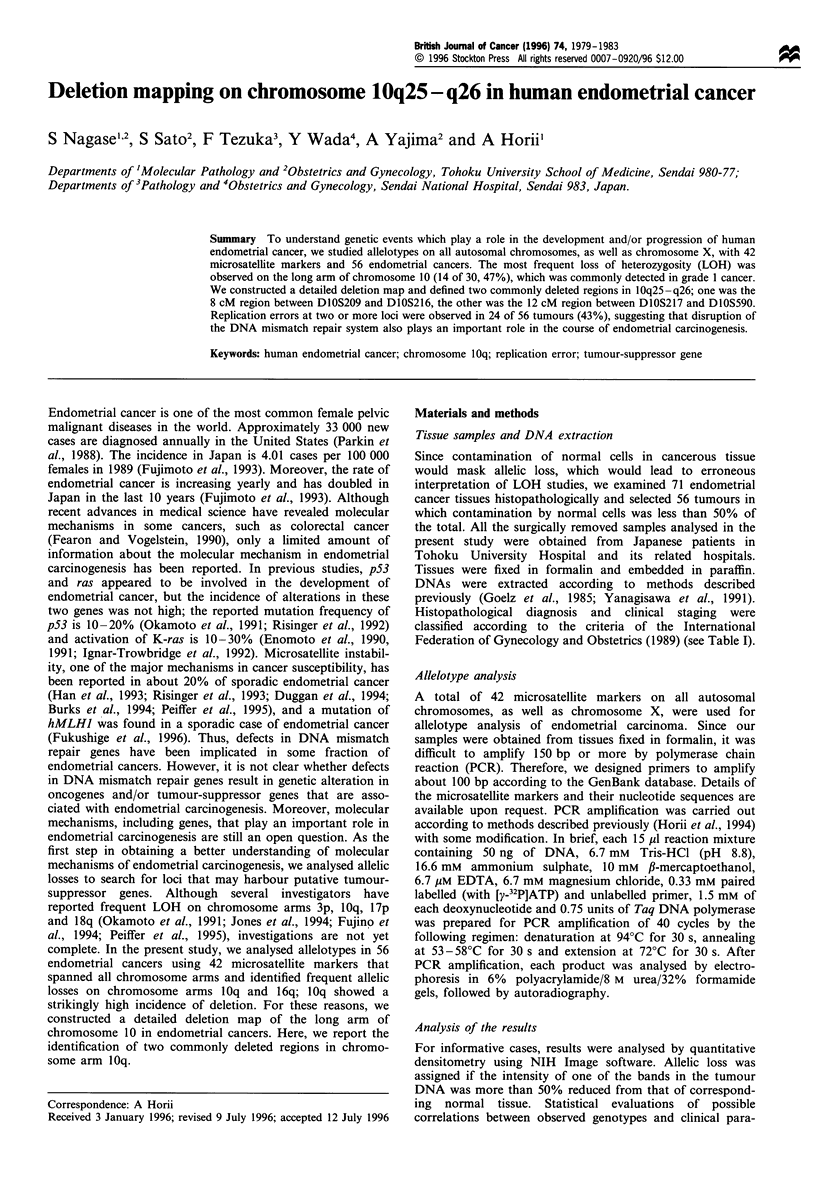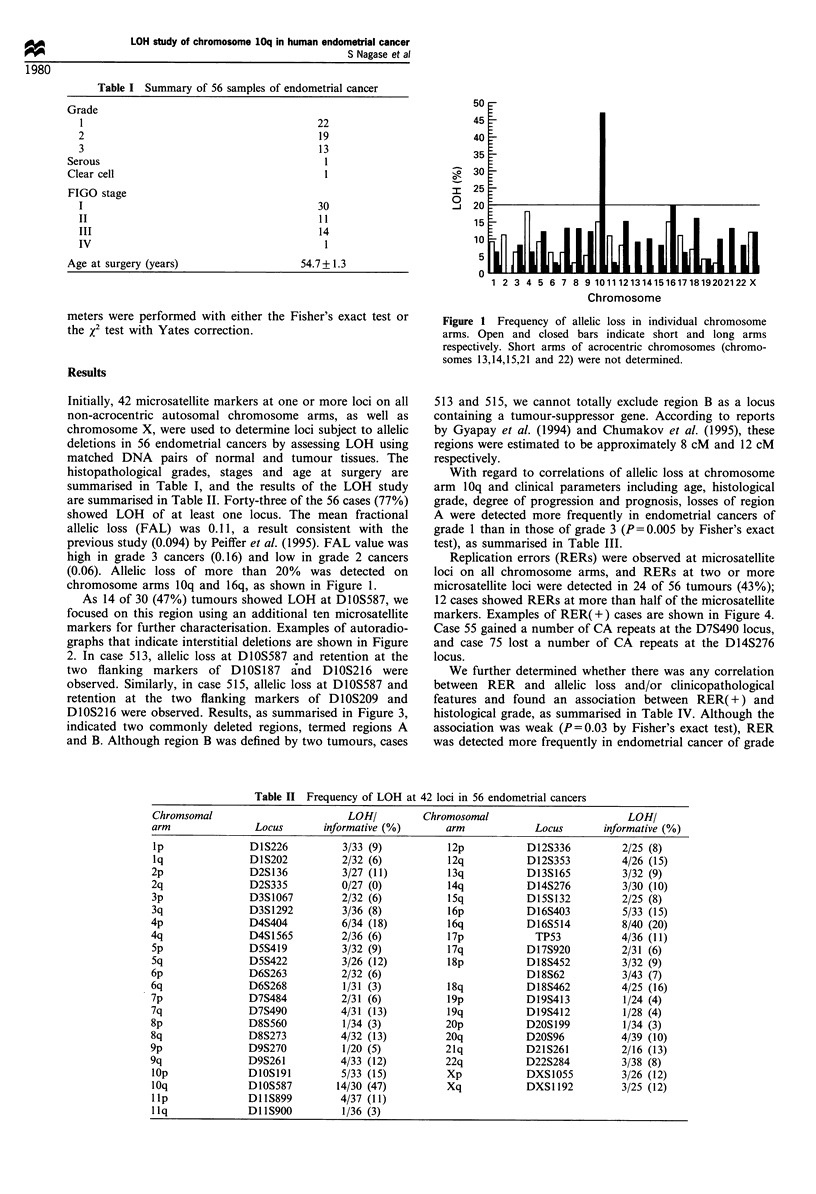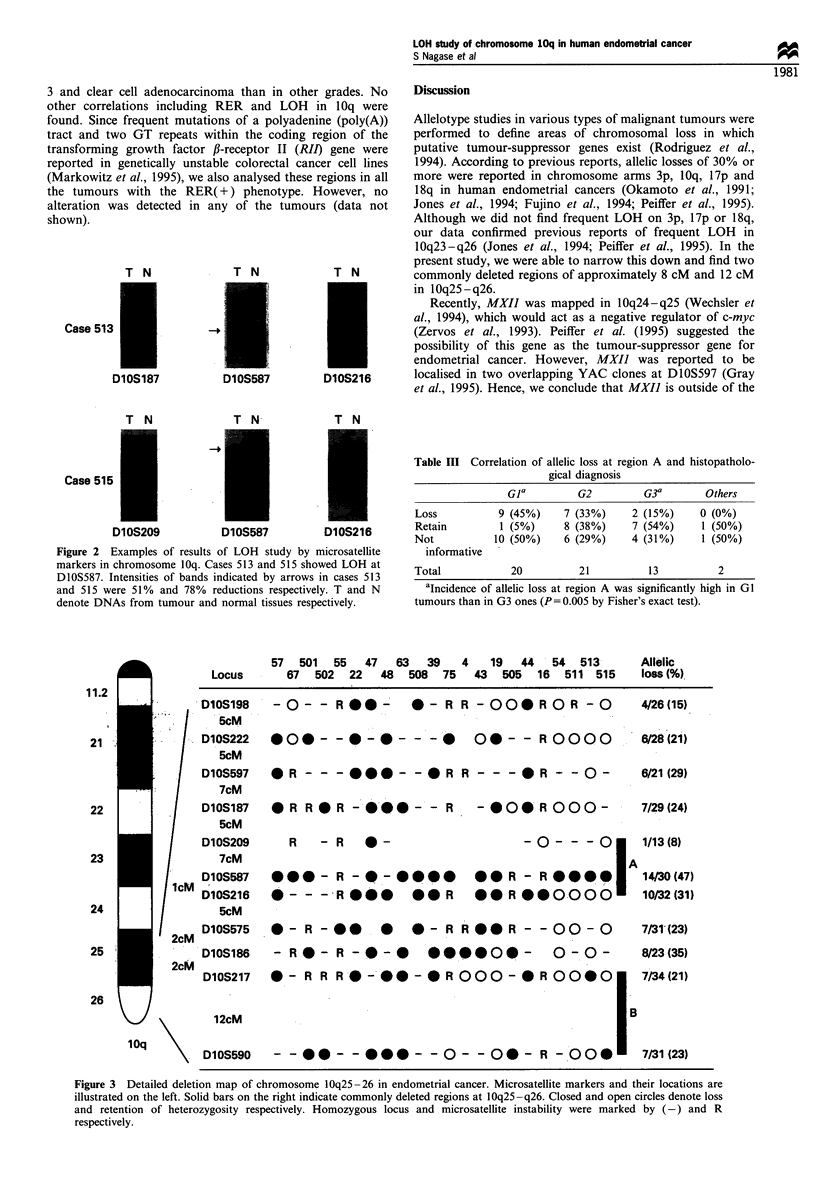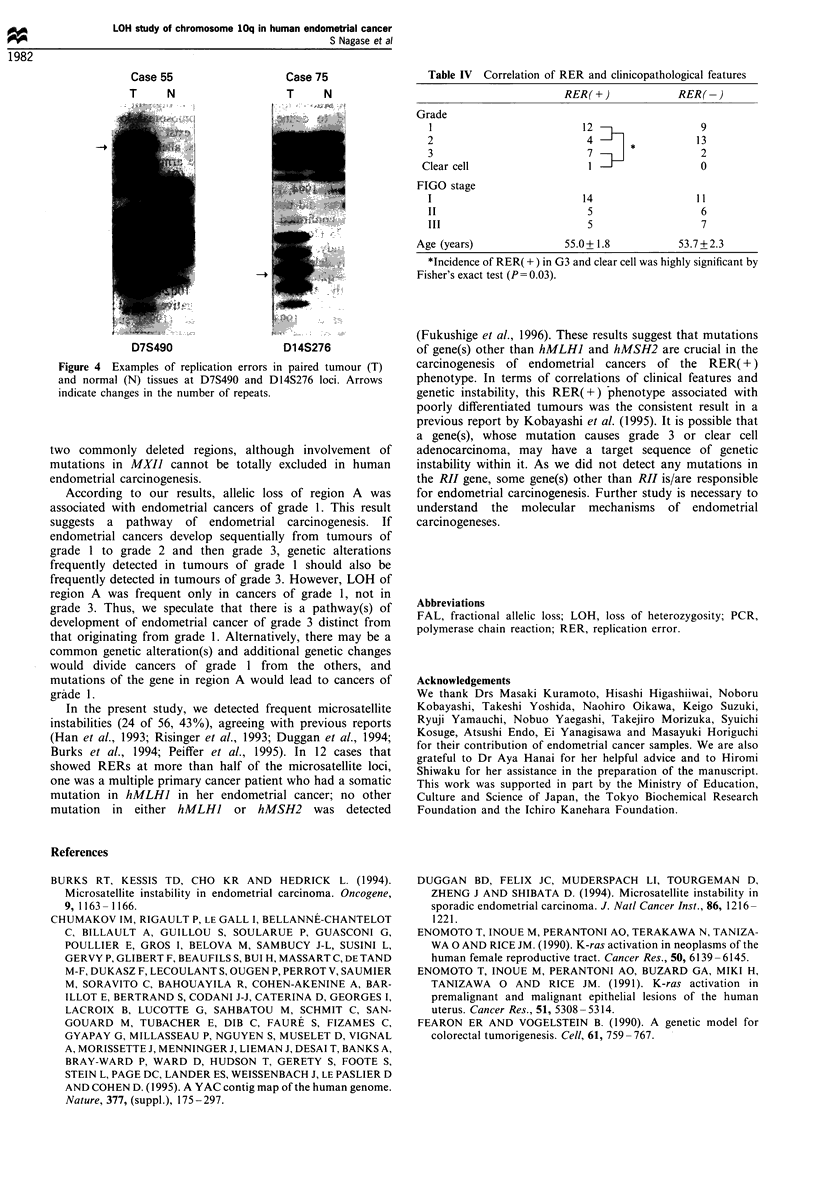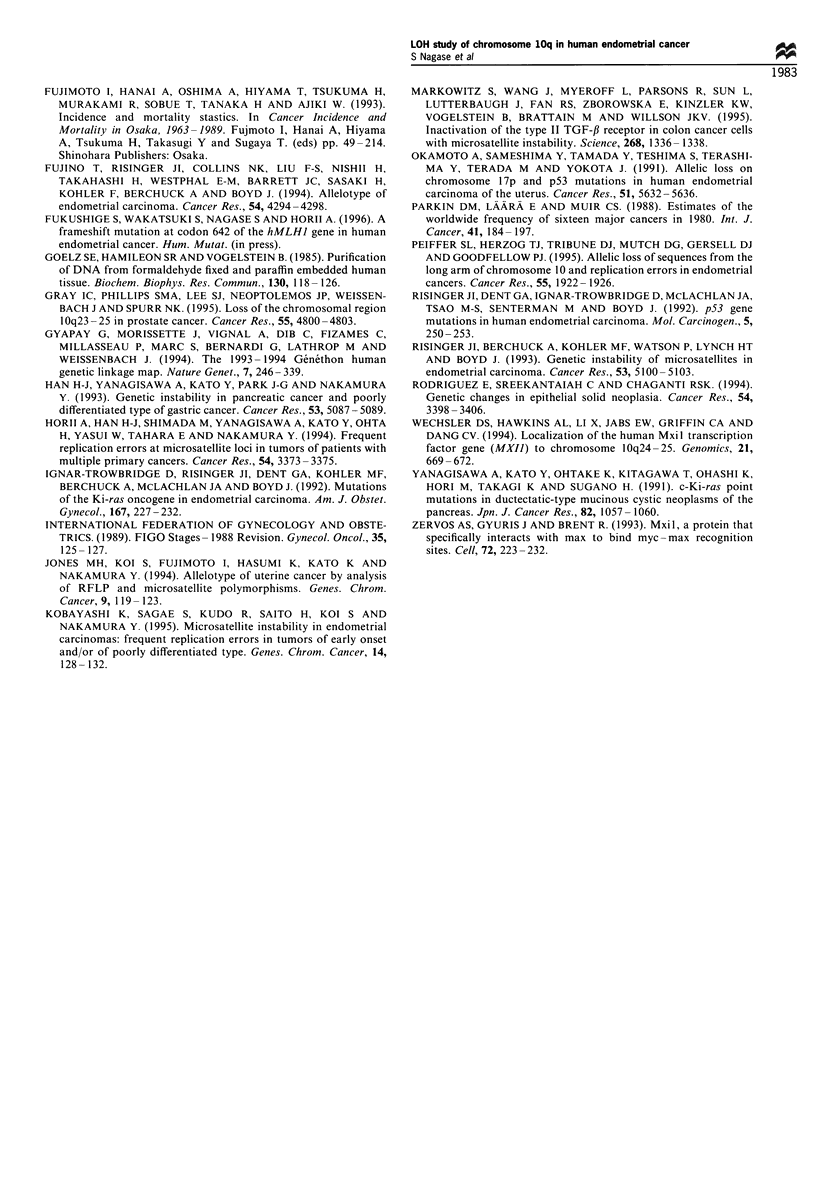# Deletion mapping on chromosome 10q25-q26 in human endometrial cancer.

**DOI:** 10.1038/bjc.1996.663

**Published:** 1996-12

**Authors:** S. Nagase, S. Sato, F. Tezuka, Y. Wada, A. Yajima, A. Horii

**Affiliations:** Department of Molecular Pathology, Tohoku University School of Medicine, Sendai, Japan.

## Abstract

**Images:**


**British Journal of Cancer (1996) 74, 1979–1983**
© 1996 Stockton Press All rights reserved 0007–0920/96 $12.00

### Deletion mapping on chromosome 10q25–q26 in human endometrial cancer

S Nagase[1,2], S Sato[2], F Tezuka[3], Y Wada[4], A Yajima[2] and A Horii[1]

*Departments of [1]Molecular Pathology and [2]Obstetrics and Gynecology, Tohoku University School of Medicine, Sendai 980-77; Departments of [3]Pathology and [4]Obstetrics and Gynecology, Sendai National Hospital, Sendai 983, Japan.*

**Summary** To understand genetic events which play a role in the development and/or progression of human endometrial cancer, we studied allelotypes on all autosomal chromosomes, as well as chromosome X, with 42 microsatellite markers and 56 endometrial cancers. The most frequent loss of heterozygosity (LOH) was observed on the long arm of chromosome 10 (14 of 30, 47%), which was commonly detected in grade 1 cancer. We constructed a detailed deletion map and defined two commonly deleted regions in 10q25–q26; one was the 8 cM region between D10S209 and D10S216, the other was the 12 cM region between D10S217 and D10S590. Replication errors at two or more loci were observed in 24 of 56 tumours (43%), suggesting that disruption of the DNA mismatch repair system also plays an important role in the course of endometrial carcinogenesis.

**Keywords:** human endometrial cancer; chromosome 10q; replication error; tumour-suppressor gene

Endometrial cancer is one of the most common female pelvic malignant diseases in the world. Approximately 33 000 new cases are diagnosed annually in the United States (Parkin *et al.*, 1988). The incidence in Japan is 4.01 cases per 100 000 females in 1989 (Fujimoto *et al.*, 1993). Moreover, the rate of endometrial cancer is increasing yearly and has doubled in Japan in the last 10 years (Fujimoto *et al.*, 1993). Although recent advances in medical science have revealed molecular mechanisms in some cancers, such as colorectal cancer (Fearon and Vogelstein, 1990), only a limited amount of information about the molecular mechanism in endometrial carcinogenesis has been reported. In previous studies, *p53* and *ras* appeared to be involved in the development of endometrial cancer, but the incidence of alterations in these two genes was not high; the reported mutation frequency of *p53* is 10–20% (Okamoto *et al.*, 1991; Risinger *et al.*, 1992) and activation of K-*ras* is 10–30% (Enomoto *et al.*, 1990, 1991; Ignar-Trowbridge *et al.*, 1992). Microsatellite instability, one of the major mechanisms in cancer susceptibility, has been reported in about 20% of sporadic endometrial cancer (Han *et al.*, 1993; Risinger *et al.*, 1993; Duggan *et al.*, 1994; Burks *et al.*, 1994; Peiffer *et al.*, 1995), and a mutation of *hMLH1* was found in a sporadic case of endometrial cancer (Fukushige *et al.*, 1996). Thus, defects in DNA mismatch repair genes have been implicated in some fraction of endometrial cancers. However, it is not clear whether defects in DNA mismatch repair genes result in genetic alteration in oncogenes and/or tumour-suppressor genes that are associated with endometrial carcinogenesis. Moreover, molecular mechanisms, including genes, that play an important role in endometrial carcinogenesis are still an open question. As the first step in obtaining a better understanding of molecular mechanisms of endometrial carcinogenesis, we analysed allelic losses to search for loci that may harbour putative tumour-suppressor genes. Although several investigators have reported frequent LOH on chromosome arms 3p, 10q, 17p and 18q (Okamoto *et al.*, 1991; Jones *et al.*, 1994; Fujino *et al.*, 1994; Peiffer *et al.*, 1995), investigations are not yet complete. In the present study, we analysed allelotypes in 56 endometrial cancers using 42 microsatellite markers that spanned all chromosome arms and identified frequent allelic losses on chromosome arms 10q and 16q; 10q showed a strikingly high incidence of deletion. For these reasons, we constructed a detailed deletion map of the long arm of chromosome 10 in endometrial cancers. Here, we report the identification of two commonly deleted regions in chromosome arm 10q.

Correspondence: A Horii
Received 3 January 1996; revised 9 July 1996; accepted 12 July 1996

### Materials and methods

### *Tissue samples and DNA extraction*

Since contamination of normal cells in cancerous tissue would mask allelic loss, which would lead to erroneous interpretation of LOH studies, we examined 71 endometrial cancer tissues histopathologically and selected 56 tumours in which contamination by normal cells was less than 50% of the total. All the surgically removed samples analysed in the present study were obtained from Japanese patients in Tohoku University Hospital and its related hospitals. Tissues were fixed in formalin and embedded in paraffin. DNAs were extracted according to methods described previously (Goelz *et al.*, 1985; Yanagisawa *et al.*, 1991). Histopathological diagnosis and clinical staging were classified according to the criteria of the International Federation of Gynecology and Obstetrics (1989) (see Table I).

### *Allelotype analysis*

A total of 42 microsatellite markers on all autosomal chromosomes, as well as chromosome X, were used for allelotype analysis of endometrial carcinoma. Since our samples were obtained from tissues fixed in formalin, it was difficult to amplify 150 bp or more by polymerase chain reaction (PCR). Therefore, we designed primers to amplify about 100 bp according to the GenBank database. Details of the microsatellite markers and their nucleotide sequences are available upon request. PCR amplification was carried out according to methods described previously (Horii *et al.*, 1994) with some modification. In brief, each 15 $\mu$l reaction mixture containing 50 ng of DNA, 6.7 mM Tris-HCl (pH 8.8), 16.6 mM ammonium sulphate, 10 mM $\beta$-mercaptoethanol, 6.7 $\mu$M EDTA, 6.7 mM magnesium chloride, 0.33 mM paired labelled (with [$\gamma$-$^{32}$P]ATP) and unlabelled primer, 1.5 mM of each deoxynucleotide and 0.75 units of *Taq* DNA polymerase was prepared for PCR amplification of 40 cycles by the following regimen: denaturation at 94°C for 30 s, annealing at 53–58°C for 30 s and extension at 72°C for 30 s. After PCR amplification, each product was analysed by electrophoresis in 6% polyacrylamide/8 M urea/32% formamide gels, followed by autoradiography.

### *Analysis of the results*

For informative cases, results were analysed by quantitative densitometry using NIH Image software. Allelic loss was assigned if the intensity of one of the bands in the tumour DNA was more than 50% reduced from that of corresponding normal tissue. Statistical evaluations of possible correlations between observed genotypes and clinical para-

Table I Summary of 56 samples of endometrial cancer

| | |
|---|---|
| Grade | |
| 1 | 22 |
| 2 | 19 |
| 3 | 13 |
| Serous | 1 |
| Clear cell | 1 |
| FIGO stage | |
| I | 30 |
| II | 11 |
| III | 14 |
| IV | 1 |
| Age at surgery (years) | 54.7±1.3 |

meters were performed with either the Fisher's exact test or the $\chi^2$ test with Yates correction.

**Figure 1** Frequency of allelic loss in individual chromosome arms. Open and closed bars indicate short and long arms respectively. Short arms of acrocentric chromosomes (chromosomes 13,14,15,21 and 22) were not determined.

### Results

Initially, 42 microsatellite markers at one or more loci on all non-acrocentric autosomal chromosome arms, as well as chromosome X, were used to determine loci subject to allelic deletions in 56 endometrial cancers by assessing LOH using matched DNA pairs of normal and tumour tissues. The histopathological grades, stages and age at surgery are summarised in Table I, and the results of the LOH study are summarised in Table II. Forty-three of the 56 cases (77%) showed LOH of at least one locus. The mean fractional allelic loss (FAL) was 0.11, a result consistent with the previous study (0.094) by Peiffer *et al.* (1995). FAL value was high in grade 3 cancers (0.16) and low in grade 2 cancers (0.06). Allelic loss of more than 20% was detected on chromosome arms 10q and 16q, as shown in Figure 1.

As 14 of 30 (47%) tumours showed LOH at D10S587, we focused on this region using an additional ten microsatellite markers for further characterisation. Examples of autoradiographs that indicate interstitial deletions are shown in Figure 2. In case 513, allelic loss at D10S587 and retention at the two flanking markers of D10S187 and D10S216 were observed. Similarly, in case 515, allelic loss at D10S587 and retention at the two flanking markers of D10S209 and D10S216 were observed. Results, as summarised in Figure 3, indicated two commonly deleted regions, termed regions A and B. Although region B was defined by two tumours, cases 513 and 515, we cannot totally exclude region B as a locus containing a tumour-suppressor gene. According to reports by Gyapay *et al.* (1994) and Chumakov *et al.* (1995), these regions were estimated to be approximately 8 cM and 12 cM respectively.

With regard to correlations of allelic loss at chromosome arm 10q and clinical parameters including age, histological grade, degree of progression and prognosis, losses of region A were detected more frequently in endometrial cancers of grade 1 than in those of grade 3 ($P=0.005$ by Fisher's exact test), as summarised in Table III.

Replication errors (RERs) were observed at microsatellite loci on all chromosome arms, and RERs at two or more microsatellite loci were detected in 24 of 56 tumours (43%); 12 cases showed RERs at more than half of the microsatellite markers. Examples of RER(+) cases are shown in Figure 4. Case 55 gained a number of CA repeats at the D7S490 locus, and case 75 lost a number of CA repeats at the D14S276 locus.

We further determined whether there was any correlation between RER and allelic loss and/or clinicopathological features and found an association between RER(+) and histological grade, as summarised in Table IV. Although the association was weak ($P=0.03$ by Fisher's exact test), RER was detected more frequently in endometrial cancer of grade

Table II Frequency of LOH at 42 loci in 56 endometrial cancers

| *Chromsomal arm* | *Locus* | *LOH/ informative* (%) | *Chromosomal arm* | *Locus* | *LOH/ informative* (%) |
|---|---|---|---|---|---|
| 1p | D1S226 | 3/33 (9) | 12p | D12S336 | 2/25 (8) |
| 1q | D1S202 | 2/32 (6) | 12q | D12S353 | 4/26 (15) |
| 2p | D2S136 | 3/27 (11) | 13q | D13S165 | 3/32 (9) |
| 2q | D2S335 | 0/27 (0) | 14q | D14S276 | 3/30 (10) |
| 3p | D3S1067 | 2/32 (6) | 15q | D15S132 | 2/25 (8) |
| 3q | D3S1292 | 3/36 (8) | 16p | D16S403 | 5/33 (15) |
| 4p | D4S404 | 6/34 (18) | 16q | D16S514 | 8/40 (20) |
| 4q | D4S1565 | 2/36 (6) | 17p | TP53 | 4/36 (11) |
| 5p | D5S419 | 3/32 (9) | 17q | D17S920 | 2/31 (6) |
| 5q | D5S422 | 3/26 (12) | 18p | D18S452 | 3/32 (9) |
| 6p | D6S263 | 2/32 (6) | | D18S62 | 3/43 (7) |
| 6q | D6S268 | 1/31 (3) | 18q | D18S462 | 4/25 (16) |
| 7p | D7S484 | 2/31 (6) | 19p | D19S413 | 1/24 (4) |
| 7q | D7S490 | 4/31 (13) | 19q | D19S412 | 1/28 (4) |
| 8p | D8S560 | 1/34 (3) | 20p | D20S199 | 1/34 (3) |
| 8q | D8S273 | 4/32 (13) | 20q | D20S96 | 4/39 (10) |
| 9p | D9S270 | 1/20 (5) | 21q | D21S261 | 2/16 (13) |
| 9q | D9S261 | 4/33 (12) | 22q | D22S284 | 3/38 (8) |
| 10p | D10S191 | 5/33 (15) | Xp | DXS1055 | 3/26 (12) |
| 10q | D10S587 | 14/30 (47) | Xq | DXS1192 | 3/25 (12) |
| 11p | D11S899 | 4/37 (11) | | | |
| 11q | D11S900 | 1/36 (3) | | | |

3 and clear cell adenocarcinoma than in other grades. No other correlations including RER and LOH in 10q were found. Since frequent mutations of a polyadenine (poly(A)) tract and two GT repeats within the coding region of the transforming growth factor β-receptor II (*RII*) gene were reported in genetically unstable colorectal cancer cell lines (Markowitz *et al*., 1995), we also analysed these regions in all the tumours with the RER(+) phenotype. However, no alteration was detected in any of the tumours (data not shown).

**Figure 2** Examples of results of LOH study by microsatellite markers in chromosome 10q. Cases 513 and 515 showed LOH at D10S587. Intensities of bands indicated by arrows in cases 513 and 515 were 51% and 78% reductions respectively. T and N denote DNAs from tumour and normal tissues respectively.

### Discussion

Allelotype studies in various types of malignant tumours were performed to define areas of chromosomal loss in which putative tumour-suppressor genes exist (Rodriguez *et al*., 1994). According to previous reports, allelic losses of 30% or more were reported in chromosome arms 3p, 10q, 17p and 18q in human endometrial cancers (Okamoto *et al*., 1991; Jones *et al*., 1994; Fujino *et al*., 1994; Peiffer *et al*., 1995). Although we did not find frequent LOH on 3p, 17p or 18q, our data confirmed previous reports of frequent LOH in 10q23–q26 (Jones *et al*., 1994; Peiffer *et al*., 1995). In the present study, we were able to narrow this down and find two commonly deleted regions of approximately 8 cM and 12 cM in 10q25–q26.

Recently, *MXI1* was mapped in 10q24–q25 (Wechsler *et al*., 1994), which would act as a negative regulator of c-*myc* (Zervos *et al*., 1993). Peiffer *et al*. (1995) suggested the possibility of this gene as the tumour-suppressor gene for endometrial cancer. However, *MXI1* was reported to be localised in two overlapping YAC clones at D10S597 (Gray *et al*., 1995). Hence, we conclude that *MXI1* is outside of the

**Table III** Correlation of allelic loss at region A and histopathological diagnosis

| | *G1*[a] | *G2* | *G3*[a] | *Others* |
|---|---|---|---|---|
| Loss | 9 (45%) | 7 (33%) | 2 (15%) | 0 (0%) |
| Retain | 1 (5%) | 8 (38%) | 7 (54%) | 1 (50%) |
| Not informative | 10 (50%) | 6 (29%) | 4 (31%) | 1 (50%) |
| Total | 20 | 21 | 13 | 2 |

[a]Incidence of allelic loss at region A was significantly high in G1 tumours than in G3 ones ($P=0.005$ by Fisher's exact test).

**Figure 3** Detailed deletion map of chromosome 10q25–26 in endometrial cancer. Microsatellite markers and their locations are illustrated on the left. Solid bars on the right indicate commonly deleted regions at 10q25–q26. Closed and open circles denote loss and retention of heterozygosity respectively. Homozygous locus and microsatellite instability were marked by (–) and R respectively.

**Figure 4** Examples of replication errors in paired tumour (T) and normal (N) tissues at D7S490 and D14S276 loci. Arrows indicate changes in the number of repeats.

two commonly deleted regions, although involvement of mutations in *MXI1* cannot be totally excluded in human endometrial carcinogenesis.

According to our results, allelic loss of region A was associated with endometrial cancers of grade 1. This result suggests a pathway of endometrial carcinogenesis. If endometrial cancers develop sequentially from tumours of grade 1 to grade 2 and then grade 3, genetic alterations frequently detected in tumours of grade 1 should also be frequently detected in tumours of grade 3. However, LOH of region A was frequent only in cancers of grade 1, not in grade 3. Thus, we speculate that there is a pathway(s) of development of endometrial cancer of grade 3 distinct from that originating from grade 1. Alternatively, there may be a common genetic alteration(s) and additional genetic changes would divide cancers of grade 1 from the others, and mutations of the gene in region A would lead to cancers of grade 1.

In the present study, we detected frequent microsatellite instabilities (24 of 56, 43%), agreeing with previous reports (Han *et al*., 1993; Risinger *et al*., 1993; Duggan *et al*., 1994; Burks *et al*., 1994; Peiffer *et al*., 1995). In 12 cases that showed RERs at more than half of the microsatellite loci, one was a multiple primary cancer patient who had a somatic mutation in *hMLH1* in her endometrial cancer; no other mutation in either *hMLH1* or *hMSH2* was detected

**Table IV** Correlation of RER and clinicopathological features

| | *RER(+)* | | *RER(−)* |
|---|---|---|---|
| Grade | | | |
| 1 | 12 | | 9 |
| 2 | 4 | * | 13 |
| 3 | 7 | | 2 |
| Clear cell | 1 | | 0 |
| FIGO stage | | | |
| I | 14 | | 11 |
| II | 5 | | 6 |
| III | 5 | | 7 |
| Age (years) | 55.0±1.8 | | 53.7±2.3 |

*Incidence of RER(+) in G3 and clear cell was highly significant by Fisher's exact test ($P=0.03$).

(Fukushige *et al*., 1996). These results suggest that mutations of gene(s) other than *hMLH1* and *hMSH2* are crucial in the carcinogenesis of endometrial cancers of the RER(+) phenotype. In terms of correlations of clinical features and genetic instability, this RER(+) phenotype associated with poorly differentiated tumours was the consistent result in a previous report by Kobayashi *et al*. (1995). It is possible that a gene(s), whose mutation causes grade 3 or clear cell adenocarcinoma, may have a target sequence of genetic instability within it. As we did not detect any mutations in the *RII* gene, some gene(s) other than *RII* is/are responsible for endometrial carcinogenesis. Further study is necessary to understand the molecular mechanisms of endometrial carcinogeneses.

### Abbreviations

FAL, fractional allelic loss; LOH, loss of heterozygosity; PCR, polymerase chain reaction; RER, replication error.

### Acknowledgements

We thank Drs Masaki Kuramoto, Hisashi Higashiiwai, Noboru Kobayashi, Takeshi Yoshida, Naohiro Oikawa, Keigo Suzuki, Ryuji Yamauchi, Nobuo Yaegashi, Takejiro Morizuka, Syuichi Kosuge, Atsushi Endo, Ei Yanagisawa and Masayuki Horiguchi for their contribution of endometrial cancer samples. We are also grateful to Dr Aya Hanai for her helpful advice and to Hiromi Shiwaku for her assistance in the preparation of the manuscript. This work was supported in part by the Ministry of Education, Culture and Science of Japan, the Tokyo Biochemical Research Foundation and the Ichiro Kanehara Foundation.

### References

BURKS RT, KESSIS TD, CHO KR AND HEDRICK L. (1994). Microsatellite instability in endometrial carcinoma. *Oncogene*, **9,** 1163–1166.

CHUMAKOV IM, RIGAULT P, LE GALL I, BELLANNÉ-CHANTELOT C, BILLAULT A, GUILLOU S, SOULARUE P, GUASCONI G, POULLIER E, GROS I, BELOVA M, SAMBUCY J-L, SUSINI L, GERVY P, GLIBERT F, BEAUFILS S, BUI H, MASSART C, DE TAND M-F, DUKASZ F, LECOULANT S, OUGEN P, PERROT V, SAUMIER M, SORAVITO C, BAHOUAYILA R, COHEN-AKENINE A, BARILLOT E, BERTRAND S, CODANI J-J, CATERINA D, GEORGES I, LACROIX B, LUCOTTE G, SAHBATOU M, SCHMIT C, SANGOUARD M, TUBACHER E, DIB C, FAURÉ S, FIZAMES C, GYAPAY G, MILLASSEAU P, NGUYEN S, MUSELET D, VIGNAL A, MORISSETTE J, MENNINGER J, LIEMAN J, DESAI T, BANKS A, BRAY-WARD P, WARD D, HUDSON T, GERETY S, FOOTE S, STEIN L, PAGE DC, LANDER ES, WEISSENBACH J, LE PASLIER D AND COHEN D. (1995). A YAC contig map of the human genome. *Nature*, **377,** (suppl.), 175–297.

DUGGAN BD, FELIX JC, MUDERSPACH LI, TOURGEMAN D, ZHENG J AND SHIBATA D. (1994). Microsatellite instability in sporadic endometrial carcinoma. *J. Natl Cancer Inst*., **86,** 1216–1221.

ENOMOTO T, INOUE M, PERANTONI AO, TERAKAWA N, TANIZAWA O AND RICE JM. (1990). K-*ras* activation in neoplasms of the human female reproductive tract. *Cancer Res*., **50,** 6139–6145.

ENOMOTO T, INOUE M, PERANTONI AO, BUZARD GA, MIKI H, TANIZAWA O AND RICE JM. (1991). K-*ras* activation in premalignant and malignant epithelial lesions of the human uterus. *Cancer Res*., **51,** 5308–5314.

FEARON ER AND VOGELSTEIN B. (1990). A genetic model for colorectal tumorigenesis. *Cell*, **61,** 759–767.

FUJIMOTO I, HANAI A, OSHIMA A, HIYAMA T, TSUKUMA H, MURAKAMI R, SOBUE T, TANAKA H AND AJIKI W. (1993). Incidence and mortality stastics. In *Cancer Incidence and Mortality in Osaka, 1963–1989*. Fujmoto I, Hanai A, Hiyama A, Tsukuma H, Takasugi Y and Sugaya T. (eds) pp. 49–214. Shinohara Publishers: Osaka.

FUJINO T, RISINGER JI, COLLINS NK, LIU F-S, NISHII H, TAKAHASHI H, WESTPHAL E-M, BARRETT JC, SASAKI H, KOHLER F, BERCHUCK A AND BOYD J. (1994). Allelotype of endometrial carcinoma. *Cancer Res.*, **54,** 4294–4298.

FUKUSHIGE S, WAKATSUKI S, NAGASE S AND HORII A. (1996). A frameshift mutation at codon 642 of the *hMLH1* gene in human endometrial cancer. *Hum. Mutat.* (in press).

GOELZ SE, HAMILEON SR AND VOGELSTEIN B. (1985). Purification of DNA from formaldehyde fixed and paraffin embedded human tissue. *Biochem. Biophys. Res. Commun.*, **130,** 118–126.

GRAY IC, PHILLIPS SMA, LEE SJ, NEOPTOLEMOS JP, WEISSENBACH J AND SPURR NK. (1995). Loss of the chromosomal region 10q23–25 in prostate cancer. *Cancer Res.*, **55,** 4800–4803.

GYAPAY G, MORISSETTE J, VIGNAL A, DIB C, FIZAMES C, MILLASSEAU P, MARC S, BERNARDI G, LATHROP M AND WEISSENBACH J. (1994). The 1993–1994 Généthon human genetic linkage map. *Nature Genet.*, **7,** 246–339.

HAN H-J, YANAGISAWA A, KATO Y, PARK J-G AND NAKAMURA Y. (1993). Genetic instability in pancreatic cancer and poorly differentiated type of gastric cancer. *Cancer Res.*, **53,** 5087–5089.

HORII A, HAN H-J, SHIMADA M, YANAGISAWA A, KATO Y, OHTA H, YASUI W, TAHARA E AND NAKAMURA Y. (1994). Frequent replication errors at microsatellite loci in tumors of patients with multiple primary cancers. *Cancer Res.*, **54,** 3373–3375.

IGNAR-TROWBRIDGE D, RISINGER JI, DENT GA, KOHLER MF, BERCHUCK A, McLACHLAN JA AND BOYD J. (1992). Mutations of the Ki-*ras* oncogene in endometrial carcinoma. *Am. J. Obstet. Gynecol.*, **167,** 227–232.

INTERNATIONAL FEDERATION OF GYNECOLOGY AND OBSTETRICS. (1989). FIGO Stages – 1988 Revision. *Gynecol. Oncol.*, **35,** 125–127.

JONES MH, KOI S, FUJIMOTO I, HASUMI K, KATO K AND NAKAMURA Y. (1994). Allelotype of uterine cancer by analysis of RFLP and microsatellite polymorphisms. *Genes. Chrom. Cancer*, **9,** 119–123.

KOBAYASHI K, SAGAE S, KUDO R, SAITO H, KOI S AND NAKAMURA Y. (1995). Microsatellite instability in endometrial carcinomas: frequent replication errors in tumors of early onset and/or of poorly differentiated type. *Genes. Chrom. Cancer*, **14,** 128–132.

MARKOWITZ S, WANG J, MYEROFF L, PARSONS R, SUN L, LUTTERBAUGH J, FAN RS, ZBOROWSKA E, KINZLER KW, VOGELSTEIN B, BRATTAIN M AND WILLSON JKV. (1995). Inactivation of the type II TGF-$\beta$ receptor in colon cancer cells with microsatellite instability. *Science*, **268,** 1336–1338.

OKAMOTO A, SAMESHIMA Y, TAMADA Y, TESHIMA S, TERASHIMA Y, TERADA M AND YOKOTA J. (1991). Allelic loss on chromosome 17p and p53 mutations in human endometrial carcinoma of the uterus. *Cancer Res.*, **51,** 5632–5636.

PARKIN DM, LÄÄRÄ E AND MUIR CS. (1988). Estimates of the worldwide frequency of sixteen major cancers in 1980. *Int. J. Cancer*, **41,** 184–197.

PEIFFER SL, HERZOG TJ, TRIBUNE DJ, MUTCH DG, GERSELL DJ AND GOODFELLOW PJ. (1995). Allelic loss of sequences from the long arm of chromosome 10 and replication errors in endometrial cancers. *Cancer Res.*, **55,** 1922–1926.

RISINGER JI, DENT GA, IGNAR-TROWBRIDGE D, McLACHLAN JA, TSAO M-S, SENTERMAN M AND BOYD J. (1992). *p53* gene mutations in human endometrial carcinoma. *Mol. Carcinogen.*, **5,** 250–253.

RISINGER JI, BERCHUCK A, KOHLER MF, WATSON P, LYNCH HT AND BOYD J. (1993). Genetic instability of microsatellites in endometrial carcinoma. *Cancer Res.*, **53,** 5100–5103.

RODRIGUEZ E, SREEKANTAIAH C AND CHAGANTI RSK. (1994). Genetic changes in epithelial solid neoplasia. *Cancer Res.*, **54,** 3398–3406.

WECHSLER DS, HAWKINS AL, LI X, JABS EW, GRIFFIN CA AND DANG CV. (1994). Localization of the human Mxi1 transcription factor gene (*MXI1*) to chromosome 10q24–25. *Genomics*, **21,** 669–672.

YANAGISAWA A, KATO Y, OHTAKE K, KITAGAWA T, OHASHI K, HORI M, TAKAGI K AND SUGANO H. (1991). c-Ki-*ras* point mutations in ductectatic-type mucinous cystic neoplasms of the pancreas. *Jpn. J. Cancer Res.*, **82,** 1057–1060.

ZERVOS AS, GYURIS J AND BRENT R. (1993). Mxi1, a protein that specifically interacts with max to bind myc–max recognition sites. *Cell*, **72,** 223–232.